# Galvanic Treatment for Comedones

**Published:** 1910-12-03

**Authors:** 


### Medicine.

### GALVANIC TREATMENT FOR COMEDONES.

It has been found, more or less by an accident, that the use of the continuous or galvanic current has a very beneficial effect in the treatment of the comedones of acne vulgaris in some cases. The fact was discovered by Dr. Blanc in France when he was using electrical treatment for the relief of insomnia. He has found that the compounds of soda which accumulate around the negative pole help to dissolve the fatty contents of the sebaceous follicles and thus get rid of the comedones. It seems likely that the cure would be temporary—that whereas the electrical current may suffice for the rapid removal of the blackheads that may be present at any one time, it will probably not prevent the reaccumulation of excess of sebum in the glands with consequent recurrence of the comedones. It is recommended that the electrode should be a broad flat one made of such size and shape as will best fit the area to be treated—forehead, cheek, or nose, as the case may be—and that it should be covered with six or seven layers of absorbent lint. It appears that sodium salicylate in a 1 per cent. solution is the best fluid with which to moisten the electrode. The negative-pole is placed upon the face, the other upon any other convenient part of the body. The current to begin with should be very weak, its strength being increased during the sitting, the maximum not exceeding 4 milliamperes and not being attained in less than two minutes; the sitting should last for five minutes unless the patient complains of unbearable pain sooner. The application may be repeated in two days' time. Some improvement is observed almost at once, and, as a rule, there is very great improvement after ten or twelve sittings. The skin becomes reddened temporarily, but this redness generally disappears in from twenty minutes to half an hour. It is claimed that no ill results accrue, the whole skin being left smooth and healthy at any rate for the time being. The treatment seems well worthy of application, and, although it may need to be repeated at intervals, it will be interesting to know how long in bad cases the improvement is found to last.

